# Evaluating significance of European-associated index SNPs in the East Asian population for 31 complex phenotypes

**DOI:** 10.1186/s12864-023-09425-y

**Published:** 2023-06-13

**Authors:** Jiahao Qiao, Yuxuan Wu, Shuo Zhang, Yue Xu, Jinhui Zhang, Ping Zeng, Ting Wang

**Affiliations:** 1 Department of Biostatistics, School of Public Health, Xuzhou Medical University, Xuzhou, 221004 Jiangsu China; 2 Center for Medical Statistics and Data Analysis, Xuzhou Medical University, Xuzhou, 221004 Jiangsu China; 3 Key Laboratory of Human Genetics and Environmental Medicine, Xuzhou Medical University, Xuzhou, 221004 Jiangsu China; 4 Key Laboratory of Environment and Health, Xuzhou Medical University, Xuzhou, 221004 Jiangsu China; 5 Engineering Research Innovation Center of Biological Data Mining and Healthcare Transformation, Xuzhou Medical University, Xuzhou, 221004 Jiangsu China

**Keywords:** Trans-ethnic genetic correlation, Genetic heterogeneity, Summary statistics, Genome-wide association study, Complex phenotype, Population transferability, Trans-ethnic false discovery rate

## Abstract

**Background:**

Genome-wide association studies (GWASs) have identified many single-nucleotide polymorphisms (SNPs) associated with complex phenotypes in the European (EUR) population; however, the extent to which EUR-associated SNPs can be generalized to other populations such as East Asian (EAS) is not clear.

**Results:**

By leveraging summary statistics of 31 phenotypes in the EUR and EAS populations, we first evaluated the difference in heritability between the two populations and calculated the trans-ethnic genetic correlation. We observed the heritability estimates of some phenotypes varied substantially across populations and 53.3% of trans-ethnic genetic correlations were significantly smaller than one. Next, we examined whether EUR-associated SNPs of these phenotypes could be identified in EAS using the trans-ethnic false discovery rate method while accounting for winner's curse for SNP effect in EUR and difference of sample sizes in EAS. We found on average 54.5% of EUR-associated SNPs were also significant in EAS. Furthermore, we discovered non-significant SNPs had higher effect heterogeneity, and significant SNPs showed more consistent linkage disequilibrium and allele frequency patterns between the two populations. We also demonstrated non-significant SNPs were more likely to undergo natural selection.

**Conclusions:**

Our study revealed the extent to which EUR-associated SNPs could be significant in the EAS population and offered deep insights into the similarity and diversity of genetic architectures underlying phenotypes in distinct ancestral groups.

**Supplementary Information:**

The online version contains supplementary material available at 10.1186/s12864-023-09425-y.

## Background

Over the last few years, large-scale genome-wide association studies (GWASs) have successfully identified hundreds of thousands of single-nucleotide polymorphisms (SNPs) associated with many complex human diseases and quantitative traits [1–4]. These discoveries considerably advance the identification of functional variation underlying phenotypes and facilitate the understanding of how SNPs affect disease risk. However, the majority of current GWASs are predominantly undertaken in homogenous populations of European (EUR) ancestry, with relatively little attention paid on other populations [5–14]. For instance, approximately 90% of participants at the discovery stage of GWASs are of EUR descent, while only 7.4% were of Asian ancestry and less than 1% are of Africans (AFR) [8]. Until recently, trans-ethnic GWASs with non-EUR descents have been increasingly conducted [15–18], revealing new novel associations in other ancestral groups including AFR [19] and East Asian (EAS) ancestries [20–26].

Those multi-ancestry GWASs found that significant SNPs identified in EUR could be discovered in other populations in the sense that they often exhibited a high consistence in effect direction and magnitude [17, 18, 27–34], indicating the same phenotypes share similar genetic component across diverse populations [32, 35–40]. However, population-specific association patterns also widely emerged, implying heterogeneous genetic architectures across diverse ancestries [22, 33, 41–51]. Furthermore, for some phenotype-associated SNPs, ancestor-relevant heterogeneity produced great differences in minor allele frequency (MAF) and linkage disequilibrium (LD) patterns; consequently, significant SNPs in one population might not be easily detected in other populations [7, 17, 51–58]. Ancestral heterogeneity was also observed for genetic architectures underlying gene expressions across diverse populations [59, 60].

Given the widespread genetic differentiation of populations between different ancestral groups [18, 61–63], the extent to which phenotype-associated SNPs identified in the European ancestry can be generalized across other populations is not completely clear [5, 64, 65]. Assessing the significance of association discoveries across diverse ancestral groups is not trivial. First, the number of SNPs is large in a typical GWAS, an extremely small significance level (e.g., 5.0 × 10^–8^) is required to avoid false positive. Current GWASs remain weak or moderate in their ability to detect associations between weakly-related SNPs and phenotypes. Limiting attention only to genome-wide significant SNPs would result in selection bias in effect estimation — a well-known phenomenon referred to as winner's curse [66, 67]. Therefore, correcting deviation of estimated effect from the true one is crucial in trans-ethnic analysis. Second, the sample size of EUR GWASs is generally several orders larger than that in non-EUR studies, which likely results in the challenge to distinguish the ancestral heterogeneity from the sample size difference. These issues make it hard to conduct a comprehensive trans-ethnic assessment of similarity and diversity of genetic components underlying phenotypes.

Previous studies investigated the reproducibility of GWAS findings at limited phenotypes or at a small group of prominent SNPs, demonstrating the similarity and diversity of related SNPs in ancestral populations [15, 29, 38, 62]. However, they often failed to take the sample size difference into account and did not correct the winner's curse. In addition, some previous studies focused primarily on trans-ethnic genetic correlation [38, 40, 68], which only quantifies the global similarity across the genome but cannot describe in detail the association pattern of individual SNPs. Overall, due to the polygenic nature of many phenotypes, it is unclear whether the previous can be generalized to other phenotypes or genome-wide significant SNPs.

To fill in the above knowledge gaps, we here analyzed 31 phenotypes with GWAS summary statistics available from the EAS and EUR populations. As large-scale GWASs continue to report index SNPs (independent variants with the lowest *P* value in significant genomic loci regions) [69, 70] and some important post-GWAS integrative analyses (e.g., polygenic score prediction [71]) also rely on them, we thus examined whether EUR-associated index SNPs could be detected to be significant in the EAS population. Note that, although index SNPs are not necessarily causal variants, our analysis is still important to understand transferability of genetic discoveries and to design powerful genomic studies in understudied ancestral groups in the future.

## Results

### Overview of employed statistical methods

We here demonstrate an overview of statistical methods applied in our analyses and give more descriptions in the Materials and Methods Section. Briefly, we analyzed a total of 31 phenotypes (i.e., 6 binary and 25 continuous) between the EAS and EUR populations (Table S1), including diseases (e.g., breast cancer (BRC) and type II diabetes (T2D)), blood cell traits (e.g., neutrophil (NEUT) and monocyte count (MONO)), lipids (e.g., high-density lipoprotein cholesterol (HDL), triglyceride (TG) and total cholesterol (TC)), and anthropometric traits such as body mass index (BMI) and height. More details regarding disease diagnosis, phenotypic definition and measurement can be found in respective original papers.

**Fig. 1:**
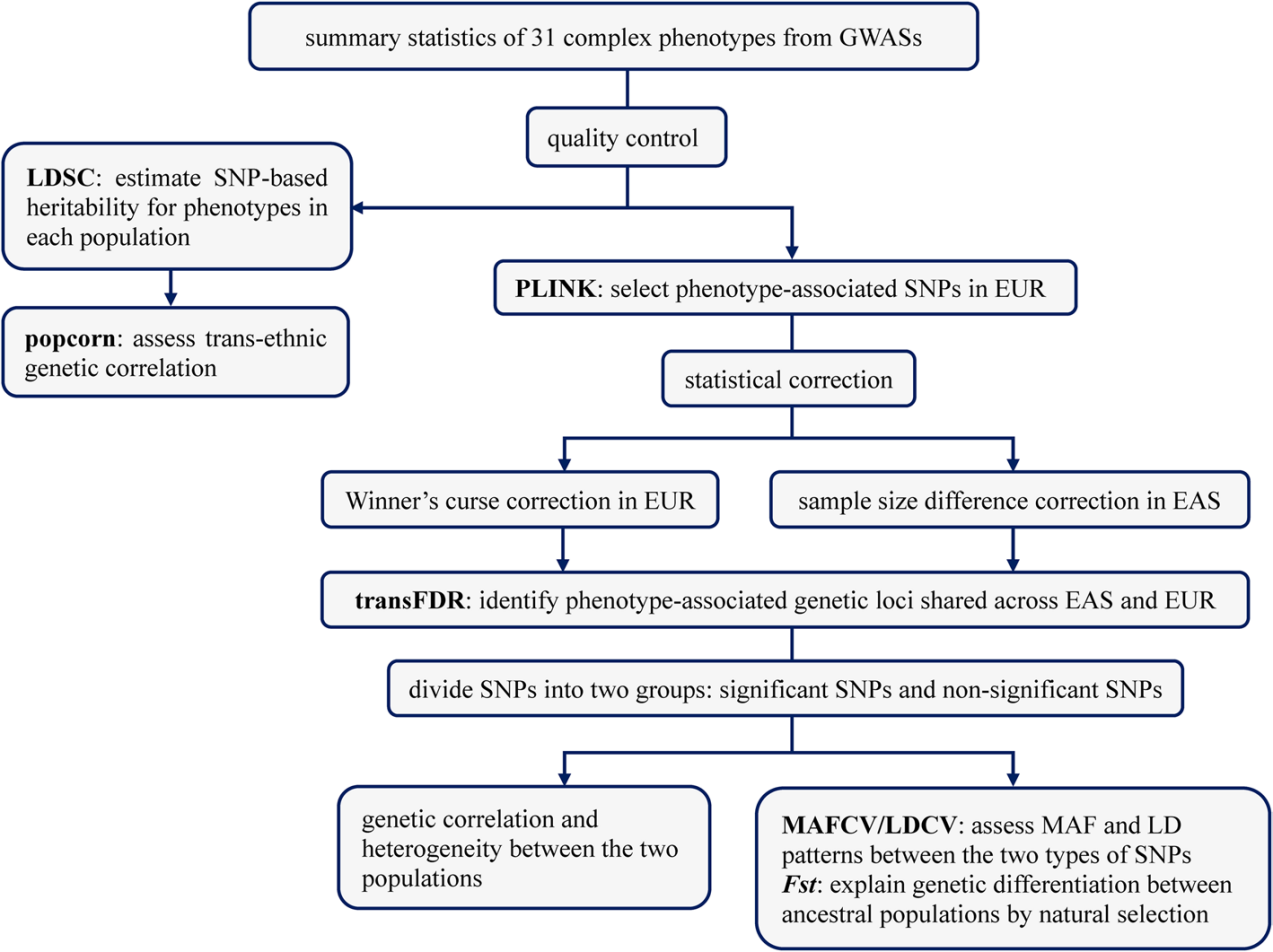
Statistical analysis framework for the theoretical and application. LDSC: LD score regression; transFDR: trans-ethnic false discovery rate; MAFCV: coefficient of variation of minor allele frequency; LDCV: coefficient of variation of LD score

We first calculated the trans-ethnic genetic correlation via popcorn [38] to examine genetic similarity and diversity of these phenotypes between the EAS and EUR populations. Then, to assess whether the genome-wide significant index SNPs discovered in the EUR population could be also detected in the EAS population, we performed the trans-ethnic false discovery rate (transFDR) method while taking winner’s curse and sample size difference into consideration [72–74]. We finally examined the heterogeneity between these significant and non-significant SNPs, assessed the difference in MAF and LD patterns by examining the coefficient of variation of LD (LDCV) or MAF (MAFCV) for the two types of SNPs, and studied whether genetic differentiation between ancestral populations could be explained by natural selection. The statistical analysis framework is shown in Fig. 1.

### Estimated heritability

We found that the estimated SNP-based heritability $({\widehat{h}}^{2})$ was highly correlated for these phenotypes across the populations (Pearson’s correlation = 0.631, *P* = 1.42 × 10^–4^) (Table 1); however, we did observe that the heritability estimates of some phenotypes varied substantially between the two populations. For instance, the heritability of TC was much greater in the EUR population (${\widehat{h}}^{2}=$ 18.6%, *se* = 3.1%) relative to that in the EAS population (${\widehat{h}}^{2}=4.2\mathrm{\%}$, *se* = 0.6%) (FDR = 3.96 × 10^–8^); conversely, the heritability of atrial fibrillation (AF) was significantly lower in the EUR population (${\widehat{h}}^{2}=1.8\mathrm{\%}$, *se* = 0.2%) than that in the EAS population (${\widehat{h}}^{2}=9.2\mathrm{\%}$, *se* = 2.4%) (FDR = 2.46 × 10^–3^). More specifically, except for rheumatoid arthritis (RA) which had ${\widehat{h}}^{2}$=13.9% (*se* = 3.9%) and 12.1% (*se* = 1.5%) in the EAS and EUR populations (FDR = 0.570), respectively, all other phenotypes showed statistically different heritability estimates between the two populations (FDR < 0.05).

**Table 1:** Estimated SNP-based heritability and trans-ethnic genetic correlation of 31 complex phenotypes analyzed in this study

Phenotype	${\widehat{h}}_{1}^{2}$(*se*_1_)	${\widehat{h}}_{2}^{2}$(*se*_2_)	${\widehat{\rho }}_{g}$(*se*)	${P}_{{\Delta h}^{2}}$	FDR
RA	0.139 (0.039)	0.121 (0.015)	0.696 (0.137)	5.55 × 10^–1^	5.70 × 10^–1^
AF	0.092 (0.024)	0.018 (0.002)	0.148 (0.065)	1.86 × 10^–3^	2.46 × 10^–3^
T2D	0.065 (0.004)	0.037 (0.002)	0.926 (0.039)	8.96 × 10^–35^	5.53 × 10^–34^
COA	0.015 (0.002)	0.045 (0.005)	0.568 (0.090)	9.14 × 10^–13^	2.42 × 10^–12^
AOA	0.015 (0.002)	0.028 (0.002)	0.526 (0.108)	2.44 × 10^–11^	6.02 × 10^–11^
BRC	0.053 (0.030)	0.106 (0.010)	0.569 (1.114)	3.89 × 10^–2^	4.36 × 10^–2^
BMI	0.120 (0.007)	0.173 (0.006)	0.844 (0.036)	3.68 × 10^–45^	4.54 × 10^–44^
height	0.321 (0.017)	0.285 (0.014)	0.864 (0.037)	2.77 × 10^–5^	4.66 × 10^–5^
DBP	0.043 (0.005)	0.101 (0.004)	0.732 (0.060)	2.85 × 10^–64^	5.27 × 10^–63^
SBP	0.053 (0.005)	0.102 (0.004)	0.709 (0.050)	3.70 × 10^–43^	3.42 × 10^–42^
PP	0.035 (0.004)	0.086 (0.003)	0.735 (0.068)	1.06 × 10^–78^	3.92 × 10^–77^
HDL	0.110 (0.018)	0.238 (0.062)	0.478 (0.340)	2.15 × 10^–2^	2.57 × 10^–2^
LDL	0.045 (0.009)	0.179 (0.036)	0.772 (0.177)	6.05 × 10^–6^	1.08 × 10^–5^
TC	0.042 (0.006)	0.186 (0.031)	0.921 (0.112)	1.82 × 10^–8^	3.96 × 10^–8^
TG	0.087 (0.027)	0.209 (0.048)	NA	NA	NA
HbA1c	0.075 (0.013)	0.037 (0.005)	0.984 (0.174)	2.98 × 10^–6^	5.80 × 10^–6^
eGFR	0.070 (0.007)	0.056 (0.003)	0.830 (0.048)	3.60 × 10^–3^	4.59 × 10^–3^
ANM	0.077 (0.010)	0.136 (0.013)	0.664 (0.091)	1.86 × 10^–9^	4.30 × 10^–9^
PLT	0.111 (0.012)	0.186 (0.016)	0.843 (0.070)	9.06 × 10^–18^	2.79 × 10^–17^
RBC	0.086 (0.010)	0.151 (0.014)	0.916 (0.057)	4.06 × 10^–25^	1.88 × 10^–24^
MCV	0.127 (0.017)	0.210 (0.033)	0.870 (0.073)	3.46 × 10^–5^	5.57 × 10^–5^
HCT	0.053 (0.006)	0.104 (0.009)	0.878 (0.082)	2.42 × 10^–27^	1.28 × 10^–26^
MCH	0.108 (0.016)	0.226 (0.037)	0.865 (0.118)	6.38 × 10^–5^	9.84 × 10^–5^
MCHC	0.037 (0.006)	0.076 (0.013)	0.837 (0.149)	6.15 × 10^–6^	1.08 × 10^–5^
HGB	0.051 (0.006)	0.109 (0.012)	0.794 (0.099)	8.22 × 10^–13^	2.34 × 10^–12^
MONO	0.054 (0.009)	0.162 (0.017)	0.804 (0.090)	2.93 × 10^–22^	1.08 × 10^–21^
NEUT	0.087 (0.012)	0.115 (0.012)	0.765 (0.064)	6.65 × 10^–4^	9.11 × 10^–4^
EO	0.056 (0.010)	0.134 (0.012)	0.761 (0.087)	2.51 × 10^–23^	1.03 × 10^–22^
BASO	0.033 (0.013)	0.058 (0.006)	0.626 (0.121)	1.58 × 10^–2^	1.95 × 10^–2^
LYMPH	0.060 (0.009)	0.139 (0.011)	0.835 (0.095)	6.67 × 10^–39^	4.94 × 10^–38^
WBC	0.070 (0.008)	0.135 (0.011)	0.752 (0.060)	3.13 × 10^–19^	1.05 × 10^–18^

${\widehat{h}}_{1}^{2}$ and ${\widehat{h}}_{2}^{2}$ are the estimated SNP-based heritability of phenotypes in the EAS and EUR populations via LDSC; *se*_1_ and *se*_2_ are the corresponding standard errors. ${\widehat{\rho }}_{g}$ is the trans-ethnic genetic correlation. ${P}_{{\Delta h}^{2}}$ denotes the *P* value available from an approximate normal test for examining the difference between ${\widehat{h}}_{1}^{2}$ and ${\widehat{h}}_{2}^{2}$.

FDR is the FDR adjusted *P* value to take the multiple-comparison issue into account. *RA* rheumatoid arthritis, *AF* atrial fibrillation, *T2D* type II diabetes, *COA* childhood-onset asthma, *AOA* adult-onset asthma, *BRC* breast cancer, *BMI* body mass index, *DBP* diastolic blood pressure, *SBP* systolic blood pressure, *PP* pulse pressure, *HDL* high-density lipoprotein cholesterol, *LDL* low-density lipoprotein cholesterol, *TC* total cholesterol, *TG* triglyceride, *HbA1c* hemoglobin A1c, *eGFR* estimated glomerular filtration rate, *ANM* age at natural non-surgical menopause, *PLT* platelet count, *RBC* red blood cell count, *MCV* mean corpuscular volume, *HCT* hematocrit, *MCH* mean corpuscular hemoglobin, *MCHC* mean corpuscular hemoglobin concentration, *HGB* hemoglobin concentration, *MONO* monocyte count, *NEUT* neutrophil count, *EO* eosinophil count, *BASO* basophil count, *LYMPH* lymphocyte count, *WBC* white blood cell count. Here, TG was excluded from our trans-ethnic genetic correlation analysis because it had an estimate larger than one

### Estimated trans-ethnic genetic correlation

The trans-ethnic genetic correlation estimate (${\widehat{\rho }}_{g})$ ranged from only 0.15 (*se* = 0.07) for AF to 0.98 (*se* = 0.17) for hemoglobin Alc (HbA1c), with an average of 0.75 across all analyzed phenotypes (Table 1). Although nearly all the trans-ethnic genetic correlations (except for BRC and HDL) were larger than zero (*H*_0_: *ρ*_*g*_ = 0) (FDR < 0.05), more than half (~ 53.3%) were significantly smaller than one (*H*_0_: *ρ*_*g*_ = 1) (FDR < 0.05), indicating there existed propound heterogeneity in genetic architecture underlying these analyzed phenotypes between the EAS and EUR populations. To examine the relation between the difference in heritability and the trans-ethnic genetic correlation, we calculated the coefficient of variation of heritability for each phenotype between the two populations, and found that greater variation of cross-population heritability appeared to lead to smaller trans-ethnic genetic correlation (Pearson’s correlation = -0.337, with a marginally significant *P* of 0.069).

### EUR-associated SNPs also detected by transFDR in the EAS population

The proportion of EUR-associated SNPs also detectable in the EAS population varied greatly among these phenotypes, ranging from 33.7% for HGB to 82.7% for AF. On average, 54.5% of phenotype-associated SNPs in the EUR population were identified also to be significant in the EAS population (FDR < 0.05). Particularly, more than half of phenotypes (~ 58.0%) showed a detection proportion larger than 50%, and the detection proportion was at least 70% for several phenotypes such as BRC, AF, RA, height, estimated glomerular filtration rate (eGFR), and age at natural non-surgical menopause (ANM). However, we did not find a significant relation between the trans-ethnic genetic correlation and SNP detection proportion across these phenotypes (*P* = 0.408). This might be due to the reason that trans-ethnic genetic correlation was an overall quantity which could not completely capture the genetic heterogeneity pattern of individual associated SNPs.

### Characteristics of EAS-associated SNPs between EAS and EUR populations

#### Marginal trans-ethnic genetic correlations of SNP effect

**Fig. 2:**
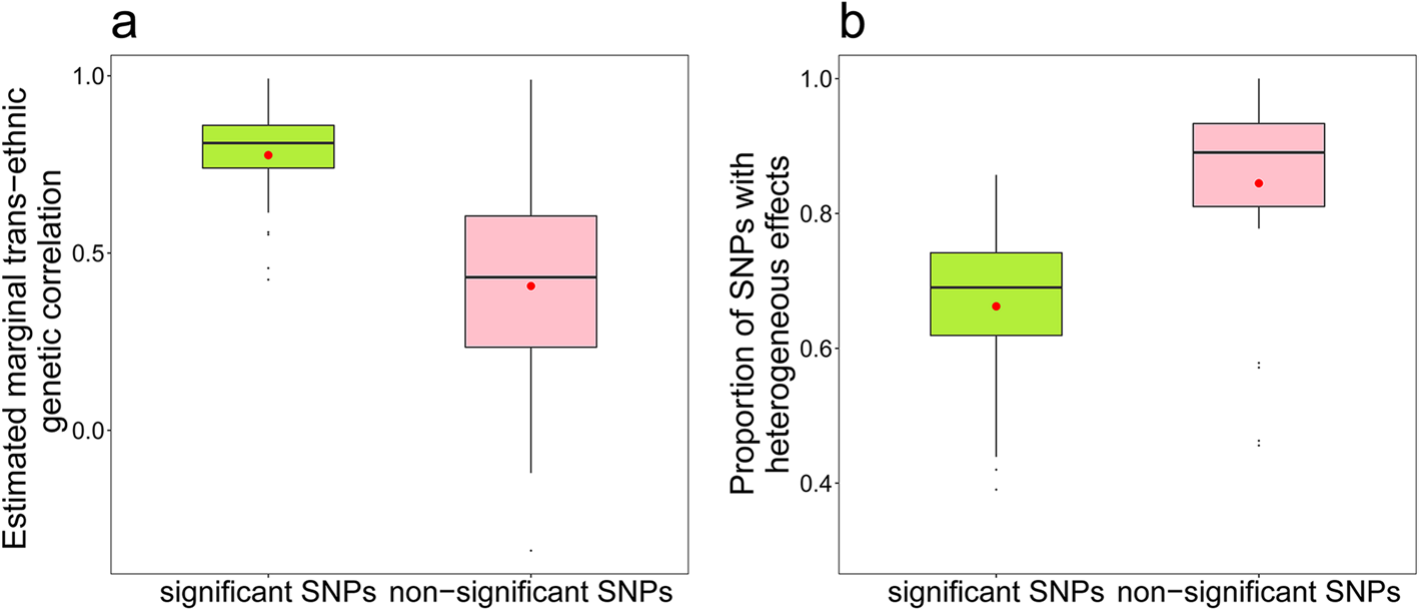
**a** Estimated marginal trans-ethnic genetic correlation across phenotypes in terms of significant and non-significant SNPs; **b** Proportion of SNPs with heterogeneous effects across phenotypes in the significant and non-significant groups

In terms of the transFDR analysis, for each phenotype we could divide these SNPs into two groups: significant or non-significant ones in the EAS population (Table 2). The significant SNPs could be also viewed as population-common variants, whereas the non-significant SNPs could be referred to as EUR-specific variants. Overall, as expected, the significant SNPs had a much greater positive correlation in effect sizes compared to these non-significant ones (${\widehat{r}}_{m}$=0.776 vs. 0.407, *P* = 4.52 × 10^–6^) (Fig. 2a). For example, ${\widehat{r}}_{m}$=0.883, 0.873 and 0.861 for these significant SNPs of mean corpuscular volume (MCV), mean corpuscular hemoglobin concentration (MCHC), and eGFR, respectively; however, the corresponding correlation was much lower for non-significant SNPs of the three phenotypes (${\widehat{r}}_{m}$=0.238, 0.520 and 0.233, respectively).

**Table 2:** Number of EUR-associated SNPs that were also discovered to be significant by transFDR in the EAS population

Phenotype	*k*	*f*_11_	*f*_01_	phenotype	*k*	*f*_11_	*f*_01_
RA	201	161	40	eGFR	344	241	103
AF	173	143	30	ANM	97	77	20
T2D	348	261	87	PLT	429	219	210
COA	199	85	114	RBC	299	155	144
AOA	91	59	32	MCV	490	263	227
BRC	251	189	62	HCT	181	71	110
BMI	1294	657	637	MCH	456	248	208
height	932	679	253	MCHC	144	74	70
DBP	1132	500	632	HGB	205	69	136
SBP	1088	523	565	MONO	374	187	187
PP	889	403	486	NEUT	200	108	92
HDL	173	105	68	EO	307	129	178
LDL	145	62	83	BASO	100	42	58
TC	174	69	105	LYMPH	287	126	161
TG	104	50	54	WBC	242	105	137
HbA1c	52	29	23				

*k* is the total number of index SNPs which were related to phenotypes in the EUR population; *f*_11_ is the number of significant SNPs identified by transFDR in the EAS population; *f*_01_ is the number of EUR-associated SNPs that were not detected to be significant in the EAS population

In addition, we found that on average 19.4% of SNPs showed opposite effects on phenotypes in the EAS and EUR populations. As projected, the proportion of SNPs with directionally discordant effects was totally smaller in the significant group compared with that in the non-significant group (9.1% vs. 31.2%, *P* = 1.29 × 10^–6^).

#### Heterogeneity in effects for significant and non-significant SNPs

Furthermore, we observed that SNPs showed evidently distinct effects even for those significant ones. In terms of the heterogeneity test, for most of analyzed phenotypes (90.3% = 28/31), we discovered that SNPs in the non-significant group had a higher degree of effect heterogeneity than those in the significant group (*P* = 2.67 × 10^–4^) (Fig. 2b). For instance, the average proportion of SNPs with heterogeneous impacts in the significant group was 66.2% across all phenotypes, compared to 79.5% in the non-significant group.

**Fig. 3:**
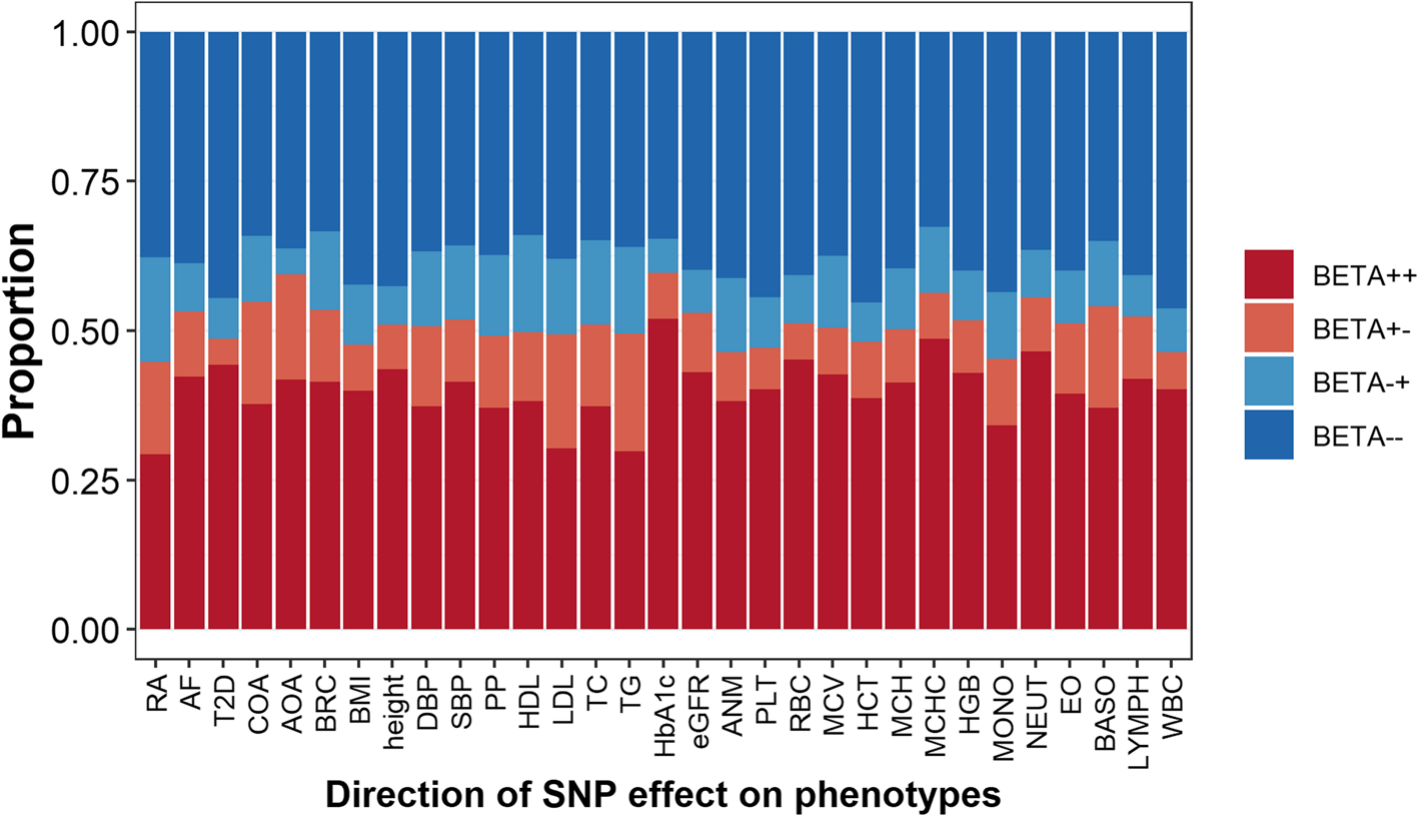
Proportion for SNPs with different effect direction between the EUR and EAS populations. *BETA*_++_ represents the proportion that SNPs had positive effects in both populations; *BETA*_+_*-* represents the proportion that SNPs had positive effect in the EAS population while negative effect in the EUR population; *BETA-*_+_ represents the proportion that SNPs had positive effect in the EUR population while negative effect in the EAS population; *BETA–* represents the proportion that SNPs had negative effects in both populations

For individual SNPs, the majority of them (80.1%) showed genetic effects with the same direction (i.e., both in positive or negative direction) on the phenotypes across the EUR and EAS populations; however, 19.9% displayed genetic effects in different directions (Fig. 3). In particular, rs57912571, associated with RA, showed the largest difference in effect (-0.892 ± 0.031 vs. 0.207 ± 0.040, *P*_diff_ < 0.001), followed by rs370433041 which was related to childhood-onset asthma (COA) (0.357 ± 0.051 vs. -0.118 ± 0.032, *P*_diff_ = 1.47 × 10^–29^) and rs79616997 which was relevant to BRC (-0.050 ± 0.007 vs. 0.460 ± 0.028, *P*_diff_ = 9.74 × 10^–95^).

#### MAF patterns for significant and non-significant SNPs

**Fig. 4:**
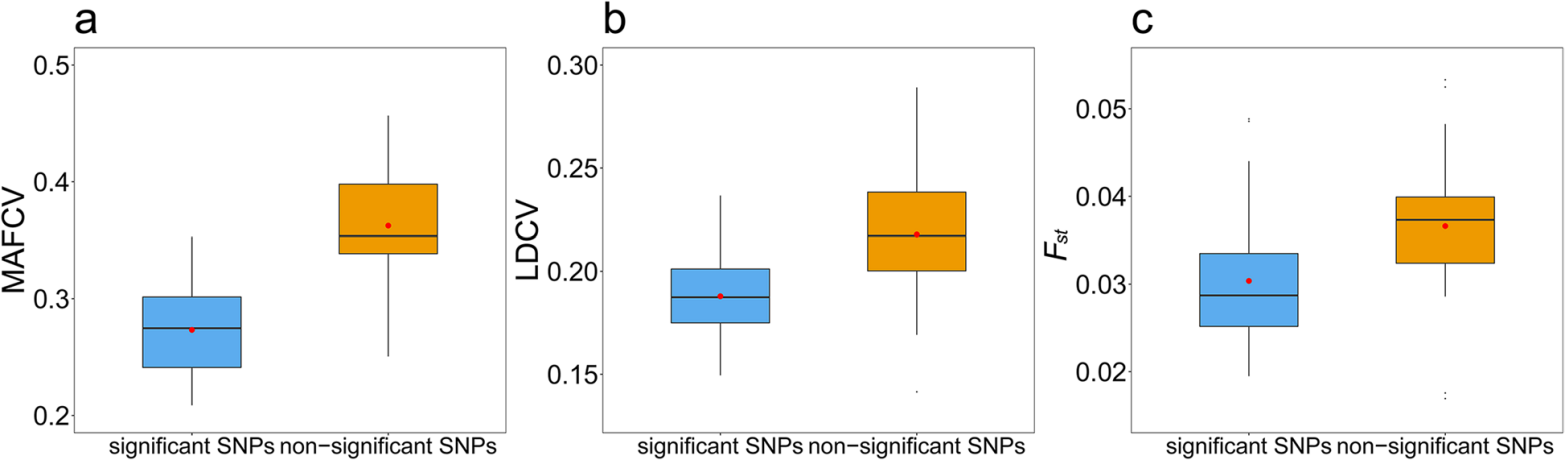
**a** MAFCV averaged across all analyzed phenotypes in the significant and non-significant groups of SNPs; **b** LDCV averaged across all analyzed phenotypes in the significant and non-significant SNPs groups; **c** *F*_*st*_ averaged across all analyzed phenotypes in the significant and non-significant groups of SNPs

MAFCV of each phenotype also showed an evident difference between the significant and non-significant SNP groups (FDR < 0.05). The average MAFCV of all phenotypes was much smaller for these significant SNPs than that for the non-significant ones (0.27 ± 0.04 vs. 0.37 ± 0.04, *P* = 4.97 × 10^–6^) (Fig. 4a). Particularly, except for MCHC and basophil count (BASO), the MAFCV of significant SNPs in all other phenotypes was smaller than that of the significant ones.

For individual SNPs, rs3001362, related to platelet count (PLT), displayed the largest MAF difference (MAF = 0.492 and 0.036 in the EUR and EAS populations, respectively), followed by rs7048601 of BMI (MAF = 0.485 and 0.069 in the EUR and EAS populations, respectively) and rs6806529 of systolic blood pressure (SBP) (MAF = 0.434 and 0.024 in the EUR and EAS populations, respectively).

#### LD score patterns for significant and non-significant SNPs

We further demonstrated that all phenotypes had a different pattern of LD scores between the significant and non-significant SNP groups (FDR < 0.05). The average LDCV of these significant SNPs was significantly smaller than that of the non-significant SNPs (0.19 ± 0.03 vs. 0.23 ± 0.04, *P* = 9.94 × 10^–4^) (Fig. 4b). Among these SNPs, rs7927898 of diastolic blood pressure (DBP) showed the greatest LD score difference (LD score = 1586.7 and 2521.2 in the EUR and EAS populations, respectively), followed by rs6990912 of T2D (LD score = 860.6 and 108.6 in the EUR and EAS populations, respectively) and rs1976672 of SBP (LD score = 893.4 and 220.6 in the EUR and EAS populations, respectively).

#### Fst patterns for significant and non-significant SNPs

Finally, we found that the average Wright’s fixation index (*F*_*st*_) of SNPs in the significant group was smaller than that in the non-significant group (0.031 ± 0.009 vs. 0.040 ± 0.016, *P* = 0.002) (Fig. 4c). Although all the 31 analyzed phenotypes were affected by natural selection (FDR < 0.05), non-significant SNPs seemed more likely to undergo natural selection. Overall, we discovered that 77.4% (= 24/31) of phenotypes had lower mean *F*_*st*_ for SNPs in the significant group.

## Discussion

### Summary of our results

In this study, we sought to evaluate the extent to which the genome-wide significant SNPs discovered in the EUR population could be also detected in the EAS population. Because the allele frequencies of phenotype-associated SNPs often varied between populations and environmental exposures could be altered [75], understanding the significance of EUR-associated SNPs in non-EUR ancestral groups thus plays a key role in uncovering the similarity and diversity of genetic architecture underlying phenotypes across distinct populations. Such knowledge is also important for identifying genetic predictors of disease risk for individuals from different ancestries, satisfying the requirement for personalized medicine and benefiting more populations from current genomics research [52].

We analyzed 31 phenotypes and found inconsistencies in heritability [60]; we also demonstrated significant but incomplete correlation among these phenotypes. These findings reflected the diversity of polygenic genetic structures across phenotypes and populations. Meanwhile, our results intuitively implied that larger difference in trans-ethnic heritability likely represented greater genetic diversity for the same phenotype in various ancestral groups. Actually, the trans-ethnic difference in heritability was not uncommon as demonstrated in previous studies [76, 77].

### Implication of our findings

There were significant genetic similarities between EUR and EAS populations, indicating by the observation that nearly all the trans-ethnic genetic correlations were larger than zero and that significant SNPs in general exhibited greater consistence in genetic influence on phenotypes than those significant only in a single population. Particularly, we found greater consistencies for some phenotypes such as T2D, which showed a larger trans-ethnic genetic correlation and a higher detection rate of EUR-associated SNPs identified to be significant in the EAS population. These high genetic consistencies imply that EAS individuals can benefit from the genomic research implemented in those of EUR ancestry; for instance, gene-based targeted treatment designed for Europeans may be also effective for non-Europeans.

Nevertheless, the SNP effects of these phenotypes had significant cross-population diversity. Moreover, even population-common SNPs showed a degree of high heterogeneity in the genetic influence of phenotypes between the EAS and EUR populations. Therefore, associated SNPs discovered in the EUR population cannot be completely and directly transferred to other populations (e.g., EAS) [15, 65]. These genetic inconsistencies offered an interpretation for the poor portability of polygenic score prediction across distinct ancestry groups [78], and further confirmed the benefit of increasing ancestry diversity in future GWASs for improvement of functional fine-mapping [42, 79, 80].

In addition, our results demonstrated that significant SNPs often displayed great consistence in allele frequency and LD pattern compared to population-specific variants, and that EUR-specific SNPs were more vulnerable to natural selection. These trans-ethnic genetic differences may be in part explained by interaction between gene–gene and gene-environment [81], which may also underlie the well-known inter-ethnic dissimilarities in prevalence or characteristics of many phenotypes [82–86].

### Strengths in this work

The trans-ethnic significance analysis of EUR-associated SNPs was complicated by inflation in effect estimates due to winner's curse in the EUR population and smaller sample size in the EAS population. The pivotal advantage of our work was to correct overestimated effects of EUR-associated SNPs and to explain difference in sample sizes of phenotypes between the EAS and EUR populations, which rendered us to perform an unbiased analysis for assessing the transportability of EUR-associated SNPs to other populations [51]. As a result, we found that a large number of SNPs could be discovered to be significant in the EAS population (i.e., population-common SNPs); however, we did observe at the same time that not all EUR-associated SNPs could be significant (i.e., EUR-specific SNPs).

### Potential limitations

Our study was not without limitation. First, we focused only on the EAS and EUR populations, which were deemed to be actually genetically similar, while the difference between AFR and non-AFR is even greater [87]; thus, generalizing our findings to other populations needs caution.

Second, our analysis only considered common SNPs (MAF > 0.01) whose origins are usually ancient, but ignored rare SNPs that are usually of recent origin. Theoretically, rare risk variants may be more likely to be population-specific and may carry greater risk effects [15]. The absence of rare risk variants likely leads us to underestimate genetic heterogeneity between the EAS and EUR populations.

Third, there possibly still existed genetic heterogeneity among individuals in diverse sub-groups in the EAS population. Consequently, the detection rate and genetic similarity analysis were likely affected by the composition of distinct individuals in the EAS GWASs.

Fourth, besides difference in sample sizes, other discrepancies in GWAS designs (e.g., phenotypic definition, statistical methods, and covariate considered) as well as in genetic architectures (e.g., polygenicity, effect size, MAF and LD) can affect the significance of EUR-associated SNPs in the EAS population. However, the design discrepancies are difficult to handle with only summary statistics, a comprehensive investigation regarding these discrepancies needs individual-level data, and is thus impeded by privacy concerns when sharing data [88]. To handle the potential discrepancy of LD in various populations, we previously conducted a gene-based replicability analysis in the EAS and EUR populations [89], where we aggregated multiple SNP-level association signs into a single gene-level association sign while taking LD into account.

Fifth, complex correlations among SNPs can bias the transFDR estimates [90]. Therefore, SNPs located within LD regions, such as in the major histocompatibility complex (MHC) region, should be excluded before performing transFDR to avoid false discoveries.

## Conclusions

Our study demonstrates the extent to which specific EUR-associated variants could be also significant in the EAS population, and offers insights into the similarity and diversity of genetic architecture underlying phenotypes in different ancestral groups.

## Materials and methods

### Summary statistics from large-scale GWASs

We yielded summary statistics of 31 phenotypes (i.e., 6 binary and 25 continuous) analyzed on EAS and EUR individuals from publicly available data portal of distinct GWAS consortia (Table S1). For summary statistics of every phenotype, we carried out the following quality control in both populations [38, 91]: (i) removed duplicated SNPs; (ii) filtered out non-biallelic SNPs; (iii) excluded SNPs with no rs labels; (iv) removed SNPs that were not genotyped in the 1000 Genomes Project or whose alleles did not match those there; (v) kept SNPs that had MAF > 0.01. We finally reserved the same set of SNPs for each phenotype in the two populations and further aligned the effect allele of SNPs across the EUR and EAS populations.

### Estimation of heritability and trans-ethnic genetic correlation

We first conducted LD score regression (LDSC) to estimate SNP-based heritability (*h*^2^) for all analyzed phenotypes in each population [92]. The LD score of SNP was calculated with genotypes of SNPs with MAF > 0.01 and the *P* value of the Hardy Weinberg equilibrium test > 1 × 10^–5^) with a 10 Mb window on 504 EAS or 503 EUR individuals from the 1000 Genomes Project [93]. To evaluate the difference in heritability, we performed the following hypothesis test for every phenotype

1 $$u\; = \;\frac{\hat{h}_{\text{eas}}^{2} \; - \;\hat{h}_{\text{eur}}^{2} }{\sqrt {{\{se(\hat{h}_{\text{eas}}^{2} )\} }^{2} \; + \;{\{ se(\hat{h}_{\text{eur}}^{2} )\} }^{2} \; - \;2\hat{\rho }_{g} \; \times \;se(\hat{h}_{\text{eas}}^{2} )\; \times \;se(\hat{h}_{\text{eur}}^{2} ) }}$$

where ${\widehat{h}}^{2}$ is the estimated heritability, *se*(${\widehat{h}}^{2}$) is the standard error, and ${\widehat{\rho }}_{g}$ denotes the trans-ethnic genetic correlation (*ρ*_*g*_) [38, 68], which is defined as the correlation between SNP effects and quantifies the extent to which the SNPs have the same or similar impacts on phenotypes across ancestral groups [40]. The *P* value of *u* in (1) could be easily obtained because *u* is normally distributed.

We estimated *ρ*_*g*_ via the popcorn method [38], with the trans-ethnic LD score of SNP calculated using genotypes of 504 EAS and 503 EUR individuals in the 1000 Genomes Project between the focal one and all the flanking ones within a 10 Mb window. Conceptually, *ρ*_*g*_ can be viewed as a trans-ethnic extension of genetic correlation of two distinct phenotypes in an ancestry-matched population to the same phenotype between continental populations. Therefore, *ρ*_*g*_ possesses its own importance and can be used to measure genetic similarity and diversity for phenotypes across various populations [38, 40].

We examined whether an estimated *ρ*_*g*_ (denoted by ${\widehat{\rho }}_{g}$) was different from zero or one using an approximate normal test

2 $$u\; = \;\frac{{\hat{\rho }_{g} }}{{se(\hat{\rho }_{g} )}}\;{\text{for}}\;H_{0} :\;\rho_{g} \; = \;0,\;{\text{or}}\; = \frac{{\hat{\rho }_{g} \; - \;1}}{{se(\hat{\rho }_{g} )}}\;{\text{for}}\;H_{0} :\;\rho_{g} \; = \;1$$

It needed to emphasize that, when estimating *h*^2^ or *ρ*_*g*_, we additionally performed another quality control for each phenotype in both populations by removing SNPs located within the major histocompatibility complex region (chr6: 28.5 Mb ~ 33.5 Mb) because of its complicated LD structure.

### Selection of phenotype-associated SNPs in the EUR population

To choose SNPs that were independently associated with phenotype in the EUR population, we applied the clumping procedure of PLINK [74] by setting the first and second significance levels of index SNPs to be 5 × 10^–8^, LD and the physical distance to be 0.01 and 1 Mb, respectively. The LD was estimated with genotypes of 503 individuals of EUR descent from the 1000 Genomes project. The number of significant SNPs ranged from 52 for HbA1c to 1,294 for BMI, with an average of 351 across phenotypes (Table S2). We extracted summary statistics of these selected SNPs for each phenotype from both populations for our subsequent analyses.

### Statistical correction of summary statistics for selected SNPs in both populations

#### Winner’s curse correction in the EUR population

As shown above, we chose phenotype-associated SNPs and estimated their effects only from the same data in the EUR population; this could cause profound selection bias and lead to the so-called issue of winner’s curse [66, 94, 95], which overestimated effects for SNPs in EUR [66, 67]. In order to adjust for such inflated genetic influence, we employed the maximum likelihood method given in [66].

3 $$\widehat{\beta }=\beta +s\times \frac{\phi \left(\beta /s-c\right)-\phi \left(-\beta /s-c\right)}{\Psi \left(\beta /s-c\right)+\Psi \left(-\beta /s-c\right)}$$

where *ϕ* is the probability density function of a standard normal variable, *Ψ* is the cumulative distribution function, $\widehat{\beta }$ is the observed marginal SNP effect, *β* is the true effect of that SNP (which is of our interest), *s* is the standard error of *β* and calculated as the average of standard error of $\widehat{\beta }$ across all selected SNPs for a given phenotype, and *c* = *Z*_1-*α*/2_ is the test statistic with *α* = 5 × 10^–8^.

We estimated *β* via a dense grid-point search strategy within the range of 95% confidence intervals for $\widehat{\beta }$. Once obtaining the estimate of *β* for each SNP, we re-computed its corresponding standard error by assuming the marginal* Z* score (and* P* value) unchanged; that is, se(*β*) = *β*/*Z*.

#### Sample size difference correction in the EAS population

In order to minimize the influence of sample size difference, we re-calculated the standard error of SNP for these EAS phenotypes using the method proposed in [96]. Specifically, for continuous phenotypes, we had

4 $$se(\hat{\beta })\; \approx \;\sqrt {\frac{1}{N\; \times \;f\; \times \;(1\; - \;f)}}$$

where $\widehat{\beta }$ indicates the marginal effect of SNP on the EAS phenotype, *f* is the MAF of SNP that would be computed with genotypes of 504 EAS individuals from the 1000 Genomes project if not offered in the original GWAS data, and *N* is the assumed sample size. To achieve our aim, we set *N* in (4) to be the sample size of the EUR phenotype. For binary phenotypes we calculated

5 $$se(\hat{\beta })\; \approx \;\sqrt {\frac{{N_{1} \; + \;N_{0} }}{{2N_{1} \; \times \;N_{0} \; \times \;f\; \times \;(1\; - \;f)}}}$$

Again, we set *N*_1_ and *N*_0_ to be the sample size of cases and controls of the EUR phenotype. For each SNP in the EAS phenotype which we kept its effect unchanged, but re-computed *Z* score and *P* value conditional on the standard error above.

### Trans-ethnic false discovery rate identifying significant associations

From a statistical perspective, under some modeling assumptions, we observe that the trans-ethnic genetic similarity analysis can be implemented with the similar principle of pleiotropic analysis for genetically correlated phenotypes [97]. In the past decade, many pleiotropy methods have been proposed [97–100]; among them, conditional FDR is a popular pleiotropy-informed approach [72, 73] and can be considered a novel generalization of the popular FDR from the single phenotype case to the same phenotype case in the trans-ethnic setting. Therefore, we referred to our used method as transFDR to distinguish itself from conditional FDR, with the code freely available at https://github.com/biostatpzeng/transFDR.

In our application framework, the null hypothesis of FDR was the absence of an association between a particular SNP and the phenotype of interest in one population. Based on this definition and the principle of FDR, transFDR is expressed as the posterior probability that a given SNP is not related to the EAS phenotype given that the observed *P* values of the phenotype in both populations are less than a predetermined threshold. Formally, transFDR is calculated as

6 $${\text{transFDR}}_{{\text{eas|eur}}} \;{ = }\;{\text{Pr}}(H_{0} |P_{{{\text{eas}}}} \; \le \;p_{{{\text{eas}}}} ,\;P_{{{\text{eur}}}} \; \le \;p_{{{\text{eur}}}} )$$

where *p*_eas_ and *p*_eur_ are the observed *P* values of the SNP for the phenotype in the two populations, respectively. Conditioning on the association observed for the EUR phenotype, we deemed a SNP to be also related to the EAS phenotype if transFDR_eas|eur_ < 0.05. It needed to highlight that transFDR was constructed for relatively independent SNPs, we thus conducted the LD pruning in PLINK to select uncorrelated index SNPs as described above. We efficiently estimated transFDR with an empirical Bayesian algorithm that was originally proposed for calculating the local FDR [101].

### Characteristics of significant SNPs between EAS and EUR populations

#### Genetic correlation and heterogeneity between the two populations

Based on the results of transFDR, for each phenotype we could divide all analyzed SNPs into two incompatible groups: (i) associated with the phenotype in both populations (i.e., significant SNPs); (ii) only associated with the EUR phenotype but not the EAS one (i.e., non-significant SNPs). In every group, we first examined the heterogeneity in genetic effect of each SNP on the phenotype

7 $$u\; = \;\frac{{\hat{\beta }_{eas} \; - \;\hat{\beta }_{eur} }}{{\sqrt {\{ se(\hat{\beta }_{eas} )\}^{2} \; + \;\{ se(\hat{\beta }_{eur} )\}^{2} \; - \;2\hat{r}_{m} \; \times \;se(\hat{\beta }_{eas} )\; \times \;se(\hat{\beta }_{eur} )} }}$$

where ${\widehat{\beta }}_{\mathrm{eas}}$ is the unadjusted marginal effect on the EAS phenotype, while ${\widehat{\beta }}_{\mathrm{eur}}$ is the bias-reduced marginal effect on the EUR phenotype, both *se*(${\widehat{\beta }}_{\mathrm{eas}}$) and *se*(${\widehat{\beta }}_{\mathrm{eur}}$) are the adjusted standard errors for ${\widehat{\beta }}_{\mathrm{eas}}$ and ${\widehat{\beta }}_{\mathrm{eur}}$, respectively; ${\widehat{r}}_{m}$ is the marginal trans-ethnic genetic correlation (*r*_*m*_) of effects for a set of independently associated SNPs [40], which, compared to the traditional Pearson’s correlation (denoted by *r*), is unbiased because it corrects the correlation attenuation phenomenon by taking the estimation error of effects into account under the framework of measurement error model [102, 103]. Like *ρ*_*g*_, which measures the global trans-ethnic genetic overlap, *r*_*m*_ is also an important index that can be applied to quantify marginal trans-ethnic genetic similarity and diversity [40].

Again, the *P* value of *u* in [7] was obtained under the normal approximation, which was further corrected for multiple comparisons. Afterwards, we were able to obtain the number of SNPs with heterogeneity in the significant and non-significant SNP groups for all analyzed phenotypes. For every phenotype, we conducted a chi-squared test to evaluate whether there was a substantial difference in the proportion of heterogeneous SNPs in the two groups.

#### LD, MAF patterns and natural selection for significant and non-significant SNPs

As significant SNPs generally showed higher consistence in genetic impact on the phenotype, a natural question was that whether these significant SNPs would also display greater similarity in LD and MAF patterns compared to non-significant ones [29]? To this goal, we examined LDCV or MAFCV for SNPs in the two groups [62]. We first calculated the LD scores for each SNP in both populations based on genotypes available from EAS (*n* = 504) or EUR (*n* = 503) individuals in the 1000 Genomes Project and then obtained their coefficient of variation across populations. In a similar way, we calculated MAFCV for every SNP between the two populations. Intuitively, we should expect to observe smaller between-population difference in LD score or MAF at significant SNPs than at non-significant ones.

We further explored whether the observed genetic differentiation in LD and MAF between significant and non-significant SNPs could be partly explained by natural selection. To this aim, we applied *F*_*st*_ to quantify the extent to which a particular SNP was under natural selection [62, 104, 105]. The *F*_*st*_ of SNPs was calculated with genotypes of 504 EAS and 503 EUR individuals from the 1000 Genomes Project.

Finally, to examine the difference in LDCV, MAFCV, or *F*_*st*_ in the two SNP groups, we carried out a two-sample Mann–Whitney U test for each phenotype. We also conducted a paired-sample McNemar test to assess the average of LDCV, MAFCV, or *F*_*st*_ across all phenotypes by simply ignoring uncertainty of the average in each SNP group.

## Supplementary Information

**Additional file 1: Table S1.** Summary information of complex phenotypes employed in the present study.

**Additional file 2: Table S2.** Summary statistics of those selected SNPs.

## Data Availability

All data generated or analyzed during this study are included in this published article and its supplementary information file.

## Abbreviations

GWASs: Genome-wide association studies
SNPs: Single-nucleotide polymorphisms
EUR: European
AFR: African
EAS: East Asian
MAF: Minor allele frequency
LD: Linkage disequilibrium
BRC: Breast cancer
T2D: Type II diabetes
NEUT: Neutrophil count
MONO: Monocyte count
HDL: High-density lipoprotein cholesterol
TG: Triglyceride
TC: Total cholesterol
BMI: Body mass index
transFDR: Trans-ethnic false discovery rate
LDCV: The coefficient of variation of LD
MAFCV: The coefficient of variation of MAF
AF: Atrial fibrillation
RA: Rheumatoid arthritis
HbA1c: Hemoglobin Alc
eGFR: Estimated glomerular filtration rate
ANM: Age at natural non-surgical menopause
MCV: Mean corpuscular volume
MCHC: Mean corpuscular hemoglobin concentration
COA: Childhood-onset asthma
BASO: Basophil count
PLT: Platelet count
SBP: Systolic blood pressure
DBP: Diastolic blood pressure
*F*_*st*_: The Wright’s fixation index
LDSC: LD score regression
