# Supplementary material for: Evaluating significance of European-associated index SNPs in the East Asian population for 31 complex phenotypes
Source: BMC Genomics. 2023 Jun 13;24:324. doi: 10.1186/s12864-023-09425-y (PMC10265896; doi:10.1186/s12864-023-09425-y)
Supplement: Supplementary file 1 — Additional file 1: Table S1. Summary information of complex phenotypes employed in the present study. [file 12864_2023_9425_MOESM1_ESM.docx]

**Supplementary File**

Table S1. Summary information of complex phenotypes employed in the present study

| no | phenotype | *k* | *n*_1_ | *n*_2_ | Reference | |
| --- | --- | --- | --- | --- | --- | --- |
|  |  |  |  |  | EAS | EUR |
| immune-related traits | | | | | | |
| 1 | RA | 4,276,771 | 15,273 | 43,290 | [[1](#_ENREF_1)] | [[1](#_ENREF_1)] |
| 2 | COA | 5,400,174 | 31,577 | 53,370 | [[2](#_ENREF_2)] | [[3](#_ENREF_3)] |
| 3 | AOA | 5,400,035 | 31,577 | 97,691 | [[2](#_ENREF_2)] | [[3](#_ENREF_3)] |
| metabolism-related traits | | | | | | |
| 4 | AF | 4,658,060 | 25,445 | 228,221 | [[4](#_ENREF_4)] | [[5](#_ENREF_5)] |
| 5 | T2D | 5,886,050 | 254,373 | 272,026 | [[6](#_ENREF_6)] | [[7](#_ENREF_7)] |
| 6 | HbA1c | 2,044,929 | 42,790 | 123,665 | [[8](#_ENREF_8)] | [[9](#_ENREF_9)] |
| 7 | DBP | 4,740,742 | 136,615 | 757,601 | [[8](#_ENREF_8)] | [[10](#_ENREF_10)] |
| 8 | SBP | 4,735,484 | 136,597 | 757,601 | [[8](#_ENREF_8)] | [[10](#_ENREF_10)] |
| 9 | PP | 4,735,721 | 136,249 | 757,601 | [[8](#_ENREF_8)] | [[10](#_ENREF_10)] |
| anthropometric traits | | | | | | |
| 10 | BMI | 1,936,663 | 158,284 | 681,275 | [[11](#_ENREF_11)] | [[12](#_ENREF_12)] |
| 11 | height | 1,899,014 | 159,095 | 693,529 | [[8](#_ENREF_8)] | [[12](#_ENREF_12)] |
| lipid traits | | | | | | |
| 12 | HDL | 1,828,662 | 70,657 | 95,123 | [[8](#_ENREF_8)] | [[13](#_ENREF_13)] |
| 13 | LDL | 1,824,860 | 72,866 | 90,422 | [[8](#_ENREF_8)] | [[13](#_ENREF_13)] |
| 14 | TC | 1,828,077 | 128,305 | 95,358 | [[8](#_ENREF_8)] | [[13](#_ENREF_13)] |
| 15 | TG | 1,825,514 | 105,597 | 91,598 | [[8](#_ENREF_8)] | [[13](#_ENREF_13)] |
| blood cell traits | | | | | | |
| 16 | PLT | 4,829,900 | 108,208 | 173,480 | [[8](#_ENREF_8)] | [[14](#_ENREF_14)] |
| 17 | RBC | 4,830,085 | 108,794 | 173,480 | [[8](#_ENREF_8)] | [[14](#_ENREF_14)] |
| 18 | MCV | 4,830,140 | 108,256 | 173,480 | [[8](#_ENREF_8)] | [[14](#_ENREF_14)] |
| 19 | HCT | 4,830,135 | 108,757 | 173,480 | [[8](#_ENREF_8)] | [[14](#_ENREF_14)] |
| 20 | MCH | 4,830,022 | 108,054 | 173,480 | [[8](#_ENREF_8)] | [[14](#_ENREF_14)] |
| 21 | MCHC | 4,830,247 | 108,728 | 173,480 | [[8](#_ENREF_8)] | [[14](#_ENREF_14)] |
| 22 | HGB | 4,830,057 | 108,769 | 173,480 | [[8](#_ENREF_8)] | [[14](#_ENREF_14)] |
| 23 | MONO | 4,830,006 | 62,076 | 173,480 | [[8](#_ENREF_8)] | [[14](#_ENREF_14)] |
| 24 | NEUT | 4,830,118 | 62,076 | 173,480 | [[8](#_ENREF_8)] | [[14](#_ENREF_14)] |
| 25 | EO | 4,830,002 | 62,076 | 173,480 | [[8](#_ENREF_8)] | [[14](#_ENREF_14)] |
| 26 | BASO | 4,829,969 | 62,076 | 173,480 | [[8](#_ENREF_8)] | [[14](#_ENREF_14)] |
| 27 | LYMPH | 4,830,004 | 62,076 | 173,480 | [[8](#_ENREF_8)] | [[14](#_ENREF_14)] |
| 28 | WBC | 4,830,062 | 107,964 | 173,480 | [[8](#_ENREF_8)] | [[14](#_ENREF_14)] |
| others | | | | | | |
| 29 | eGFR | 5,035,780 | 143,658 | 765,348 | [[8](#_ENREF_8)] | [[15](#_ENREF_15)] |
| 30 | ANM | 2,081,697 | 43,861 | 69,360 | [[8](#_ENREF_8)] | [[16](#_ENREF_16)] |
| 31 | PCA | 5,143,263 | 20,562 | 137,462 | [[17](#_ENREF_17)] | [[18](#_ENREF_18)] |

Note: *k* is the number of SNPs shared by the EAS and EUR GWASs, *n*_1_ and *n*_2_ are the effective sample sizes of EAS and EUR, which is the original sample size for continuous phenotypes and is the effective sample size for binary phenotypes computed via 4/(1/*n*_case_+1/*n*_control_) [[19](#_ENREF_19)].

Table S2. Summary statistics of those selected SNPs

**References**

1. Okada Y, Wu D, Trynka G, Raj T, Terao C, Ikari K, Kochi Y, Ohmura K, Suzuki A, Yoshida S: **Genetics of rheumatoid arthritis contributes to biology and drug discovery**. *Nature* 2014, **506**(7488):376-381.

2. Ishigaki K, Akiyama M, Kanai M, Takahashi A, Kawakami E, Sugishita H, Sakaue S, Matoba N, Low S-K, Okada Y *et al*: **Large-scale genome-wide association study in a Japanese population identifies novel susceptibility loci across different diseases**. *Nat Genet* 2020.

3. Ferreira MAR, Mathur R, Vonk JM, Szwajda A, Brumpton B, Granell R, Brew BK, Ullemar V, Lu Y, Jiang Y *et al*: **Genetic Architectures of Childhood- and Adult-Onset Asthma Are Partly Distinct**. *Am J Hum Genet* 2019, **104**(4):665-684.

4. Low S-K, Takahashi A, Ebana Y, Ozaki K, Christophersen IE, Ellinor PT, Consortium AF, Ogishima S, Yamamoto M, Satoh M *et al*: **Identification of six new genetic loci associated with atrial fibrillation in the Japanese population**. *Nat Genet* 2017, **49**(6):953-958.

5. Nielsen JB, Thorolfsdottir RB, Fritsche LG, Zhou W, Skov MW, Graham SE, Herron TJ, McCarthy S, Schmidt EM, Sveinbjornsson G *et al*: **Biobank-driven genomic discovery yields new insight into atrial fibrillation biology**. *Nat Genet* 2018, **50**(9):1234-1239.

6. Spracklen CN, Horikoshi M, Kim YJ, Lin K, Bragg F, Moon S, Suzuki K, Tam CHT, Tabara Y, Kwak S-H *et al*: **Identification of type 2 diabetes loci in 433,540 East Asian individuals**. *Nature* 2020.

7. Mahajan A, Taliun D, Thurner M, Robertson NR, Torres JM, Rayner NW, Payne AJ, Steinthorsdottir V, Scott RA, Grarup N *et al*: **Fine-mapping type 2 diabetes loci to single-variant resolution using high-density imputation and islet-specific epigenome maps**. *Nat Genet* 2018, **50**(11):1505-1513.

8. Kanai M, Akiyama M, Takahashi A, Matoba N, Momozawa Y, Ikeda M, Iwata N, Ikegawa S, Hirata M, Matsuda K *et al*: **Genetic analysis of quantitative traits in the Japanese population links cell types to complex human diseases**. *Nat Genet* 2018, **50**(3):390-400.

9. Wheeler E, Leong A, Liu C-T, Hivert M-F, Strawbridge RJ, Podmore C, Li M, Yao J, Sim X, Hong J *et al*: **Impact of common genetic determinants of Hemoglobin A1c on type 2 diabetes risk and diagnosis in ancestrally diverse populations: A transethnic genome-wide meta-analysis**. *PLoS Med* 2017, **14**(9):e1002383.

10. Evangelou E, Warren HR, Mosen-Ansorena D, Mifsud B, Pazoki R, Gao H, Ntritsos G, Dimou N, Cabrera CP, Karaman I *et al*: **Genetic analysis of over 1 million people identifies 535 new loci associated with blood pressure traits**. *Nat Genet* 2018, **50**(10):1412-1425.

11. Akiyama M, Okada Y, Kanai M, Takahashi A, Momozawa Y, Ikeda M, Iwata N, Ikegawa S, Hirata M, Matsuda K *et al*: **Genome-wide association study identifies 112 new loci for body mass index in the Japanese population**. *Nat Genet* 2017, **49**(10):1458-1467.

12. Yengo L, Sidorenko J, Kemper KE, Zheng Z, Wood AR, Weedon MN, Frayling TM, Hirschhorn J, Yang J, Visscher PM *et al*: **Meta-analysis of genome-wide association studies for height and body mass index in ~700000 individuals of European ancestry**. *Hum Mol Genet* 2018, **27**(20):3641-3649.

13. Willer CJ, Schmidt EM, Sengupta S, Peloso GM, Gustafsson S, Kanoni S, Ganna A, Chen J, Buchkovich ML, Mora S *et al*: **Discovery and refinement of loci associated with lipid levels**. *Nat Genet* 2013, **45**(11):1274-1283.

14. Astle WJ, Elding H, Jiang T, Allen D, Ruklisa D, Mann AL, Mead D, Bouman H, Riveros-Mckay F, Kostadima MA *et al*: **The Allelic Landscape of Human Blood Cell Trait Variation and Links to Common Complex Disease**. *Cell* 2016, **167**(5):1415-1429.

15. Wuttke M, Li Y, Li M, Sieber KB, Feitosa MF, Gorski M, Tin A, Wang L, Chu AY, Hoppmann A *et al*: **A catalog of genetic loci associated with kidney function from analyses of a million individuals**. *Nat Genet* 2019, **51**(6):957-972.

16. Day FR, Ruth KS, Thompson DJ, Lunetta KL, Pervjakova N, Chasman DI, Stolk L, Finucane HK, Sulem P, Bulik-Sullivan B *et al*: **Large-scale genomic analyses link reproductive aging to hypothalamic signaling, breast cancer susceptibility and BRCA1-mediated DNA repair**. *Nat Genet* 2015, **47**(11):1294-1303.

17. Mancuso N, Rohland N, Rand KA, Tandon A, Allen A, Quinque D, Mallick S, Li H, Stram A, Sheng X *et al*: **The contribution of rare variation to prostate cancer heritability**. *Nat Genet* 2016, **48**(1):30-35.

18. Schumacher FR, Al Olama AA, Berndt SI, Benlloch S, Ahmed M, Saunders EJ, Dadaev T, Leongamornlert D, Anokian E, Cieza-Borrella C *et al*: **Association analyses of more than 140,000 men identify 63 new prostate cancer susceptibility loci**. *Nat Genet* 2018, **50**(7):928-936.

19. Willer CJ, Li Y, Abecasis GR: **METAL: fast and efficient meta-analysis of genome-wide association scans**. *Bioinformatics* 2010, **26**:2190-2191.
